# Supplementary material for: Adenosine Increases the Immunosuppressive Capacity of Cervical Cancer Cells by Increasing PD-L1 Expression and TGF-β Production through Its Interaction with A2AR/A2BR
Source: Pharmaceuticals (Basel). 2024 Mar 19;17(3):397. doi: 10.3390/ph17030397 (PMC10974506; doi:10.3390/ph17030397)
Supplement: Supplementary file 1 [file pharmaceuticals-17-00397-s001.zip › pharmaceuticals-2865423-supplementary.pdf]

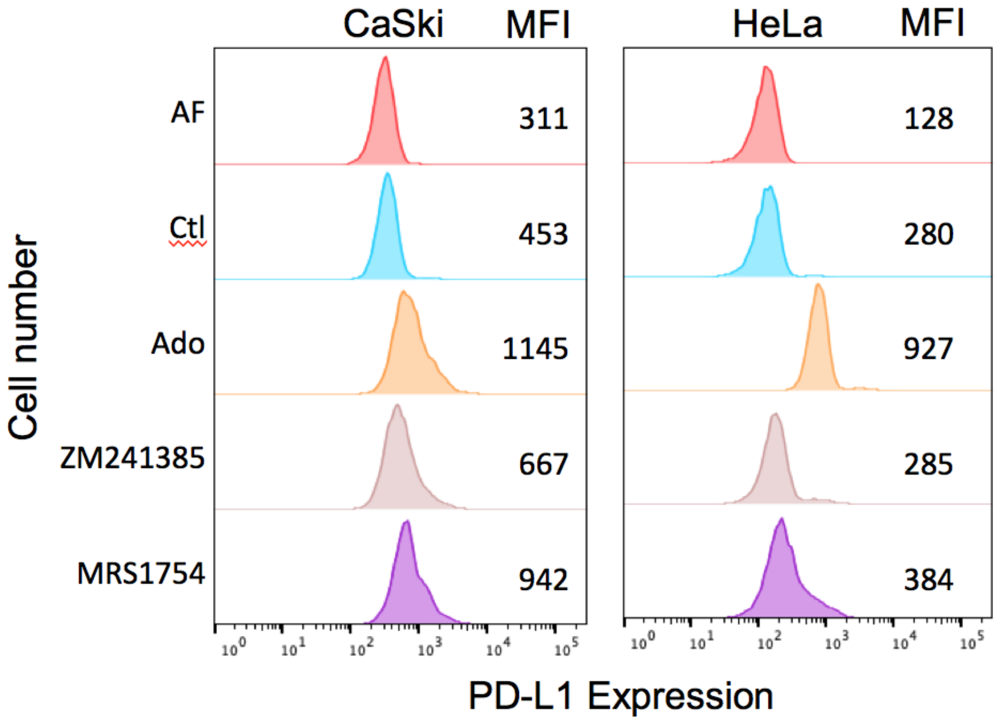

Figure S1. Blockade of $A_{2A}R$ and $A_{2B}R$ reversed the Ado-mediated induction of PD-L1 expression in CeCa cells. CaSki and HeLa cells (1×10^5 each) were first incubated for 30 min in the presence of 10 μM ZM241385 and MRS1754, which are selective antagonists of $A_{2A}R$ and $A_{2B}R$, respectively. Then, the cells were cultured for 72 h in the presence or absence of 1 mM Ado. Cells that were cultured in the absence of both Ado and AR antagonists served as a control (Ctl). PD-L1 expression was analyzed via flow cytometry. Representative data from three independent experiments are shown. MFI, mean fluorescence intensity.

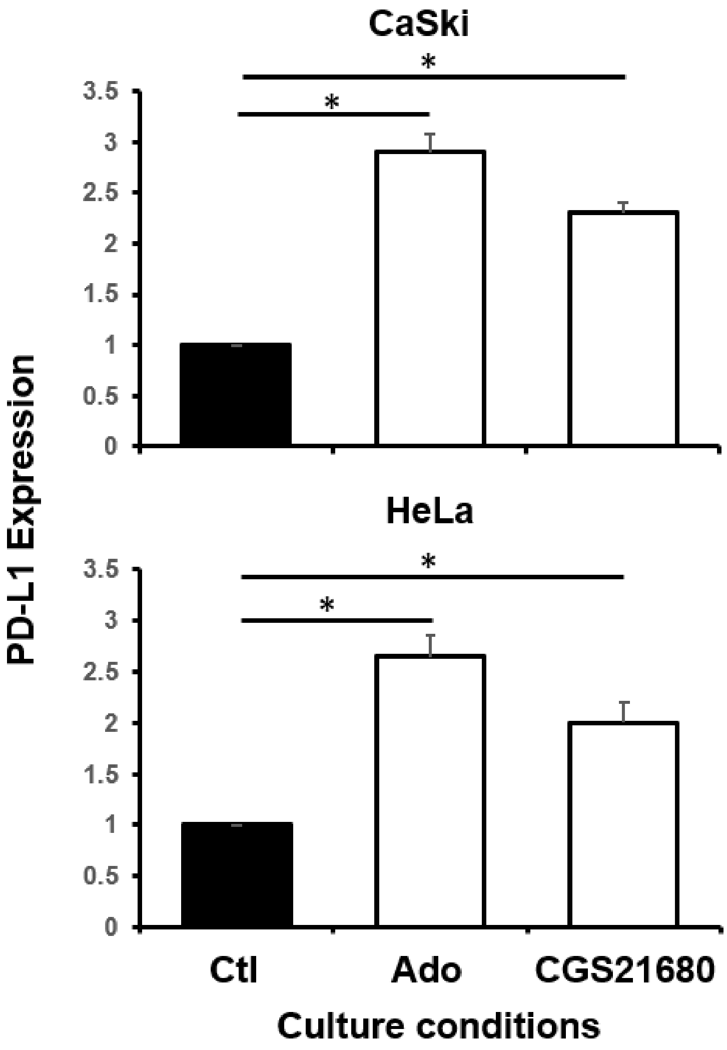

Figure S2. The selective A2 adenosine receptor agonist CGS21680 induces PD-L1 expression on CeCa cells. CaSki or HeLa cells were cultured for 72 h in the presence of 1 mM Ado or 10 mM CGS21680. Cells that were cultured in the absence of both Ado and CGS21680 served as controls (black bars). The statistical significance was calculated using the one way ANOVA (nonparametric) test. Representative data from three independent experiments (means ± SEMs) are shown. * $p < 0.001$.

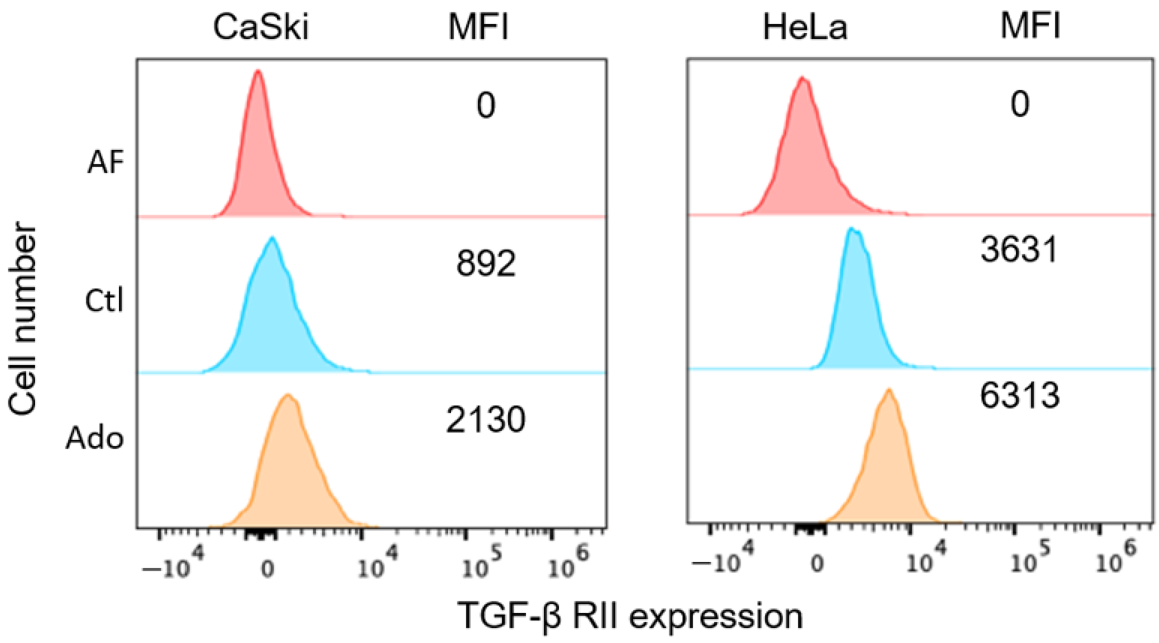

Figure S3. Effect of Ado on the expression of TGF-βRII in CeCa cells. CaSki and HeLa cells (1×10^5 each) were cultured for 72 h in the presence or absence of 1 mM Ado, after which the expression of TGF-βRII was analyzed via flow cytometry. Representative data from three independent experiments are shown. MFI, mean fluorescence intensity.
